# A novel amino acid site of N protein could affect the PRRSV-2 replication by regulating the viral RNA transcription

**DOI:** 10.1186/s12917-022-03274-9

**Published:** 2022-05-11

**Authors:** Hua Deng, Ning Xin, Fancong Zeng, Feng Wen, Heyou Yi, Chunquan Ma, Shujian Huang, Guihong Zhang, Yao Chen

**Affiliations:** 1 School of Life Science and Engineering, Foshan University, Foshan, 528000 People’s Republic of China; 2 MOA Key Laboratory of Animal Vaccine Development, Ministry of Agriculture, College of Veterinary Medicine, South China Agricultural University, Guangzhou, 510642 People’s Republic of China

**Keywords:** Porcine reproductive and respiratory syndrome virus (PRRSV), Nucleocapsid protein, Replication ability, IL-10

## Abstract

**Background:**

Finding the key amino acid sites that could affect viral biological properties or protein functions has always been a topic of substantial interest in virology. The nucleocapsid (N) protein is one of the principal proteins of the porcine reproductive and respiratory syndrome virus (PRRSV) and plays a vital role in the virus life cycle. The N protein has only 123 or 128 amino acids, some of key amino acid sites which could affect the protein functions or impair the viral biological characteristics have been identified. In this research, our objective was to find out whether there are other novel amino acid sites of the N protein can affect N protein functions or PRRSV-2 replication.

**Results:**

In this study, we found mutated the serine^78^ and serine ^99^of the nucleocapsid (N) protein can reduce the N-induced expression of IL-10 mRNA; Then, by using reverse genetics system, we constructed and rescued the mutant viruses, namely, A78 and A99.The IFA result proved that the mutations did not affect the rescue of the PRRSV-2. However, the results of the multistep growth kinetics and qPCR assays indicated that, compared with the viral replication ability, the titres and gRNA levels of A78 were significantly decreased compared with the wild-type. Further study showed that a single amino acid change from serine to alanine at position 78 of the N protein could abrogates the level of viral genomic and subgenomic RNAs. It means the mutation could significant decrease the viral replication efficiency in vitro.

**Conclusions:**

Our results suggest that the serine^78^ of N protein is a key site which could affect the N protein function and PRRSV replication ability.

**Supplementary Information:**

The online version contains supplementary material available at 10.1186/s12917-022-03274-9.

## Background

Porcine reproductive and respiratory syndrome (PRRS) is one of the most important infectious diseases in the swine industry worldwide [1]. The pathogen causing PRRS, the porcine reproductive and respiratory syndrome virus (PRRSV), was first reported in the United States in 1987. Now, it has spread around the world and caused substantial economic losses [2].

PRRSV is a positive-sense RNA virus and belongs to the *Arterivirus* family; the genome is approximately 15 kb [3]. Based on their genetic diversity, PRRSVs can be divided into PRRSV-1 (European strains) and PRRSV-2 (North American strains) [4]. Due to the high variability of the PRRSV nucleotide sequences, PRRSVs can be further classified into nine lineages [5]. Because no effective drugs or treatments are available, vaccines remain the principal means of protecting pigs from PRRSV infections [6–8]. However, most of the vaccines are attenuated vaccines derived from the wild-type virus, which were repeatedly passaged in the MA-104 cell line or in Marc-145 cells, such as JXA1-R and TJM [9, 10]. However, the mechanism of its diminished virulence has not been elucidated. Many studies have suggested that this method of reducing virulence is unstable and carries some risks [4, 11]. Due to the highly mutable nature of the PRRSVs, improving safety is one of the primary goals of PRRSV vaccines [10]. Therefore, better methods to attenuate virulence are needed.

Previous reports have shown that the mutation of some key amino acid sites, such as the amino acid at position 154 of the envelope glycoprotein of the Duck Tembusu virus and the amino acid at position 614 of the spike protein of SARS-CoV-2, may change virulence or viral replication ability [12, 13]. PRRSV has at least 23 proteins, including 16 non-structural proteins and 7 structural proteins. Previous reports indicated that if aspartic^185^ of the Nsp4 was mutated, the viral capacity to antagonize IFN-I expression of Nsp4 would decline and result in a slower replication efficiency for the PRRSV [14]. The serine^519^, threonine^544^, threonine^586^ and serine^592^ of the Nsp9 are vital amino acids for controlling the PRRSV replication rate and could change PRRSV virulence [15, 16].

The nucleocapsid (N) protein is the most abundant protein in the PRRSV and plays a vital role in the viral life cycle, including regulating cytokine levels in the host, connecting with other viral proteins and combining with viral RNA [17]. As a protein with 123 or 128 amino acids, a mutation to an amino acid site may alter the functions of the N protein or affect viral replication ability and virulence [18, 19].

In this study, we showed that the S78A or S99A mutations can affect the N protein’s ability to regulate IL-10 mRNA expression levels; Using a reverse genetics system and IFA, we found that mutated position 78 or 99 on the N protein from serine to alanine did not affect the rescue of PRRSV. However, compared with wild-type, the A78 replication ability in vitro were decrease; Further investigations found that a single amino acid change from serine to alanine at position 78 of the N protein can impair viral subgenomic RNA (sgmRNA) transcriptional levels. Our study proves that the serine^78^ is a novel amino acid site that can regulate N protein functions and PRRSV-2 replication.

## Results

### Serine^78^ and Serine^99^ of N protein could affect N-induced IL-10 mRNA

**Fig. 1:**
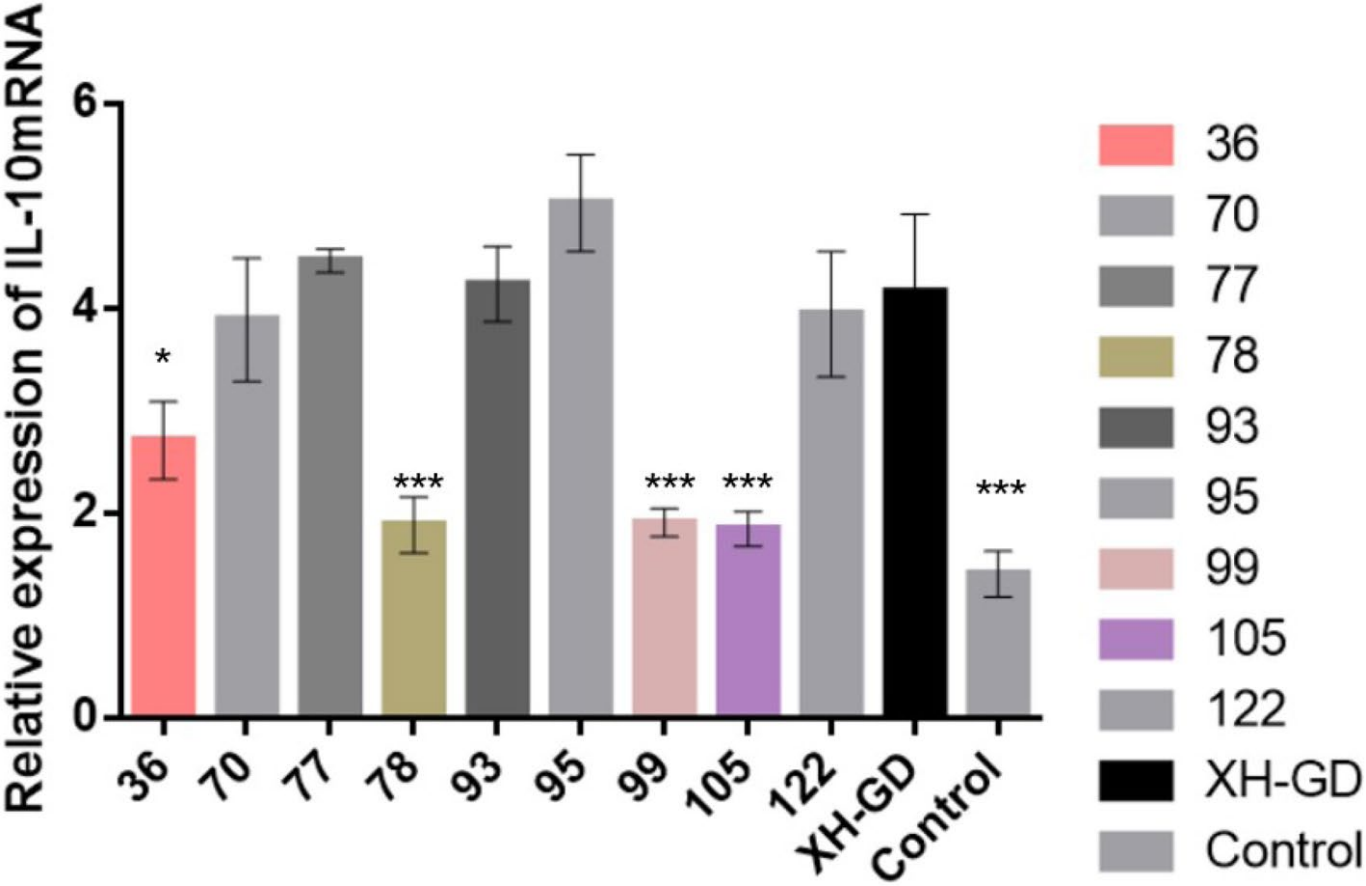
Substituting amino acids 36, 78, 99 and 105 of the N protein from serine to alanine decreased the IL-10 mRNA level. The plasmids were transfected into PAM cells (3D4/2). After 48 h, the samples were collected for qPCR analysis. Compared with β-actin, the relative IL-10 mRNA level was determined. Each data point represents the mean value of triplicates (*, *P* < 0.05; **, *P* < 0.01; ***, *P* < 0.001 in comparison with the XH-GD group)

**Fig. 2:**
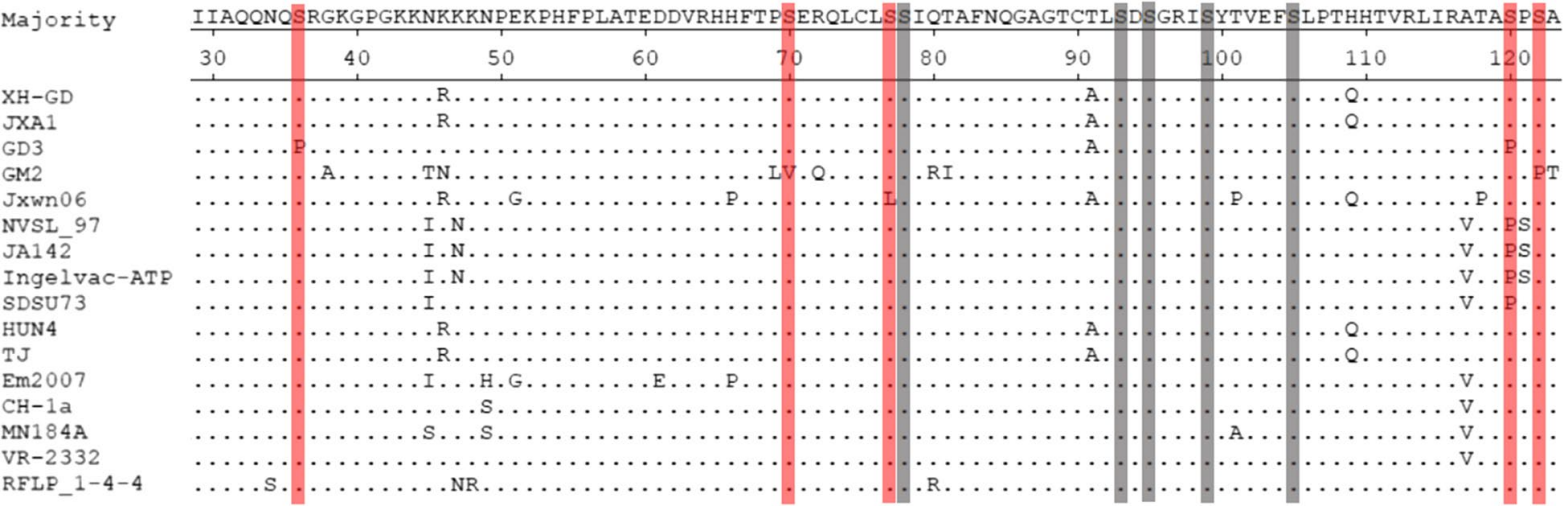
Sequence analysis for the PRRSV-2 N protein. Fifteen isolates were found in the National Center for Biotechnological Information (NCBI) database. The conserved serine sites (78, 93, 95, 99 and 105) and the non-conserved serine sites (36, 70, 77, 120 and 122) are shown in grey and red

Regulating host cytokines, such as IL-10 or IRF3, is one of the major functions of the N protein [20, 21]. N protein has ten serine sites, three of them (serine^36^, serine^105^ and serine^120^) have confirmed to be involved in regulating IL-10 mRNA or IL-10 expression [18, 19, 22]. These results indicated that the serine of N protein could affect the N-induced expression of IL-10. We wondered whether the other serine sites have similar functions. Therefore, we mutated the remaining serine sites, constructed ten plasmids and transfected them into PAMs (3D4/2). After 48 h, the cells were collected for qPCR. As shown in Fig. 2, compared with the other plasmids, the IL-10 mRNA levels of pCA-78 and pCA-99 were significantly reduced, as were those of pCA-105 and pCA-36. This means that mutations in serine ^78^ and serine ^99^ could affect N-induced IL-10 mRNA expression (Fig. 1).

### Mutation of serine^78^ and serine^99^ did not affect viral viability or infectivity

**Fig. 3:**
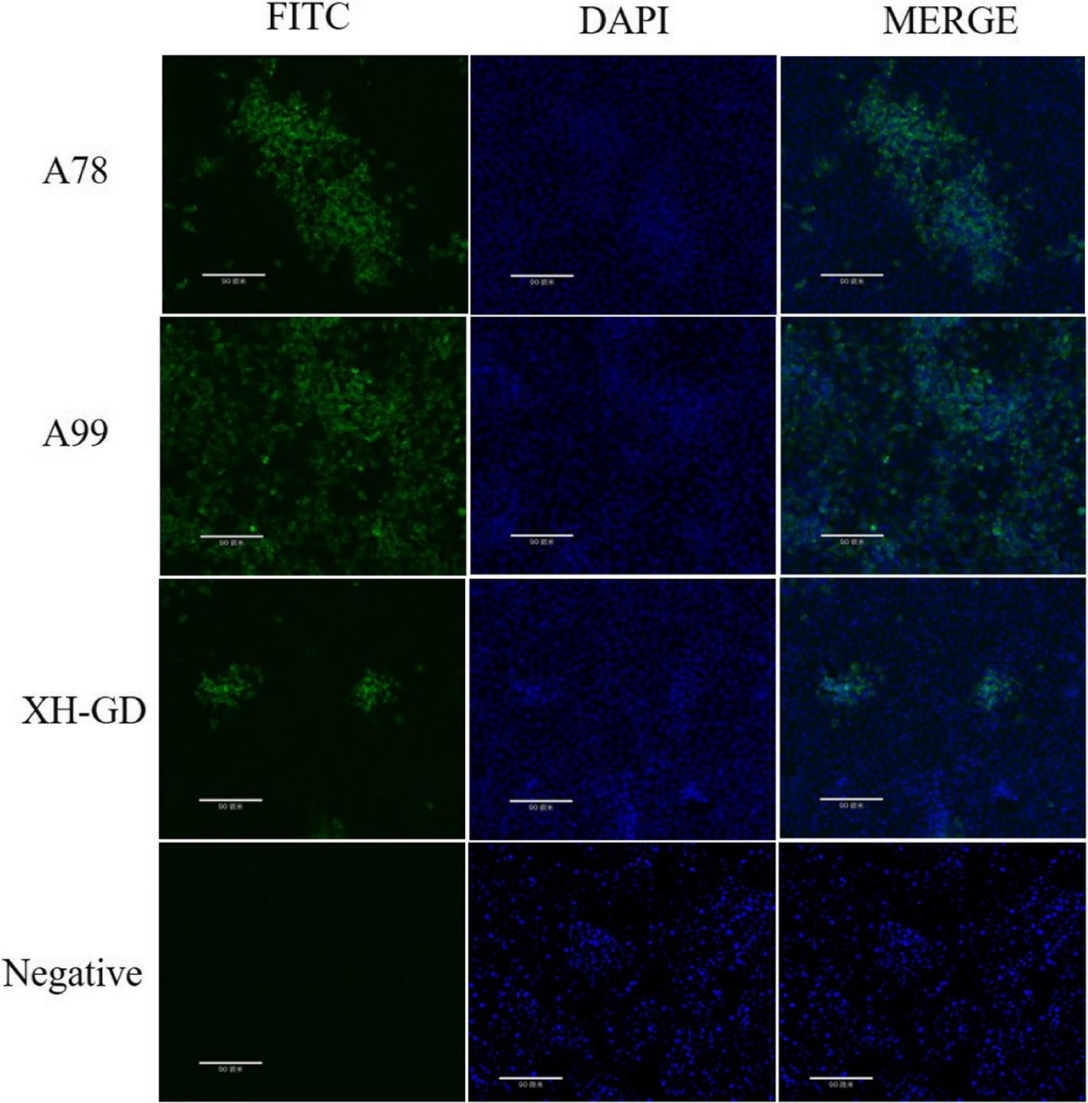
IFA results. The Marc-145 cells were infected with the PRRSV at 0.1MOI. At 48 hpi, the infected cells were fixed with 4% paraformaldehyde and PBS. Then, the cells were incubated with the N monoclonal antibody, followed by goat anti-mouse IgG (H + L), which was modified by fluorescein isothiocyanate (FITC). The nuclei were stained with DAPI. (Scale bars are 90 um)

The nucleotide analysis showed that the serine^78^ and serine^99^ of N protein were conserved among different PRRSV subgroup (Fig. 2). Hence, we wondered whether the serine^78^ and serine^99^ could affect viral viability or infectivity. In order to verify this hypothesis, a series of infectious clones were constructed and transfected into Marc-145 cells. The rescued virus genomes were confirmed by sequencing and passage three times. IFA was used to examine whether the mutated viruses could be rescued. The results showed that none of the mutations affected PRRSV viability or infectivity (Fig. 3). It is suggested that the mutations did not affect virus replication and assembly.

### Mutation of serine^78^ could affect the in Vitro replication efficiency of PRRSV

**Fig. 4:**
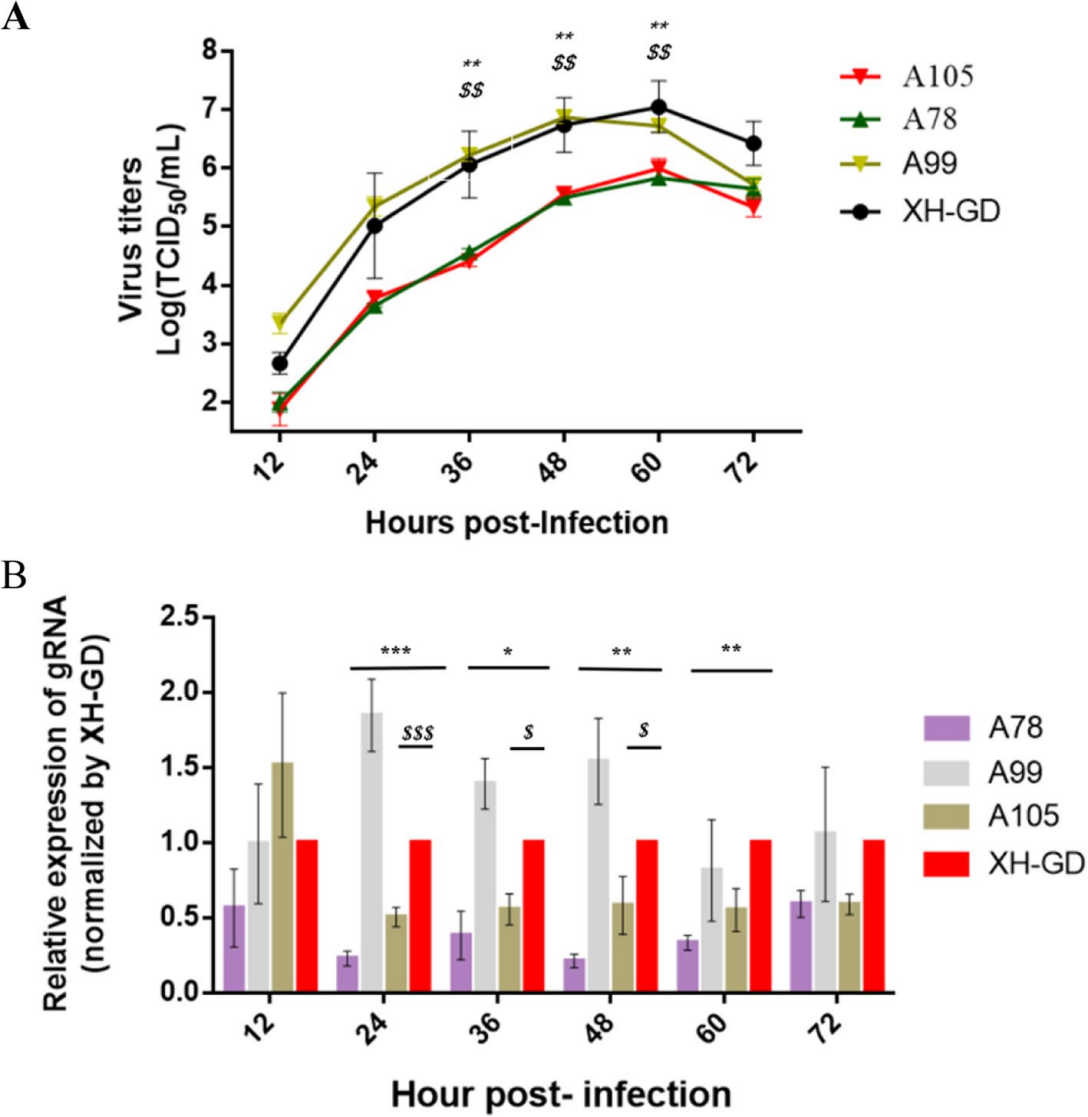
Growth characterization of the mutated viruses. **A**: The multistep kinetics of the mutated viruses. Marc-145 cells were infected with PRRSV at an MOI of 0.1. The supernatants were collected at various time points and titrated. The viral titres were calculated by the Reed-Muench method; **B**: The gRNA qPCR results of the mutated viruses. Viral RNA was extracted and subjected to qPCR analysis, and the change in gRNA levels compared with β-actin was determined. Each data point represents the mean value of triplicates. (*, *P* < 0.05; **, *P* < 0.01; ***, *P* < 0.001 in comparison with the XH-GD group). (^$^: WT vs. A78; ^*^: WT vs. A105)

Previous studies have shown that mutating the serine^105^ and serine^120^ of the N protein can reduce viral replication and virulence [18]. However, mutations to serine ^36^ and serine ^122^ do not change the viral replication ability [18, 19, 22, 23]. Therefore, we questioned whether the serine^78^ or serine ^99^ of the N protein could change PRRSV replication efficiency. Therefore, the A78, A99, A105 and wild-type strains were used to compare viral replicability in Marc-145 cellsby multistep growth kinetics. The results showed that the curves for A99 was similar to the XH-GD at six time points. However, the titre for A78, likely A105, was significantly reduced from 36 to 60hpi compared with XH-GD (Fig. 4A).

For further quantitative analysis of the viruses, qPCR was performed to compare the expression levels of the viral gRNA. The results showed the viral gRNA expression level of A99 was higher than the parent virus at 24 hpi. The gRNA expression levels for A78 were lower than that of XH-GD from 24 to 60 hpi; This means that the replication ability of A78, which was similar to that of A105, was slower than that of XH-GD in vitro (Fig. 4B).

**Fig. 5:**
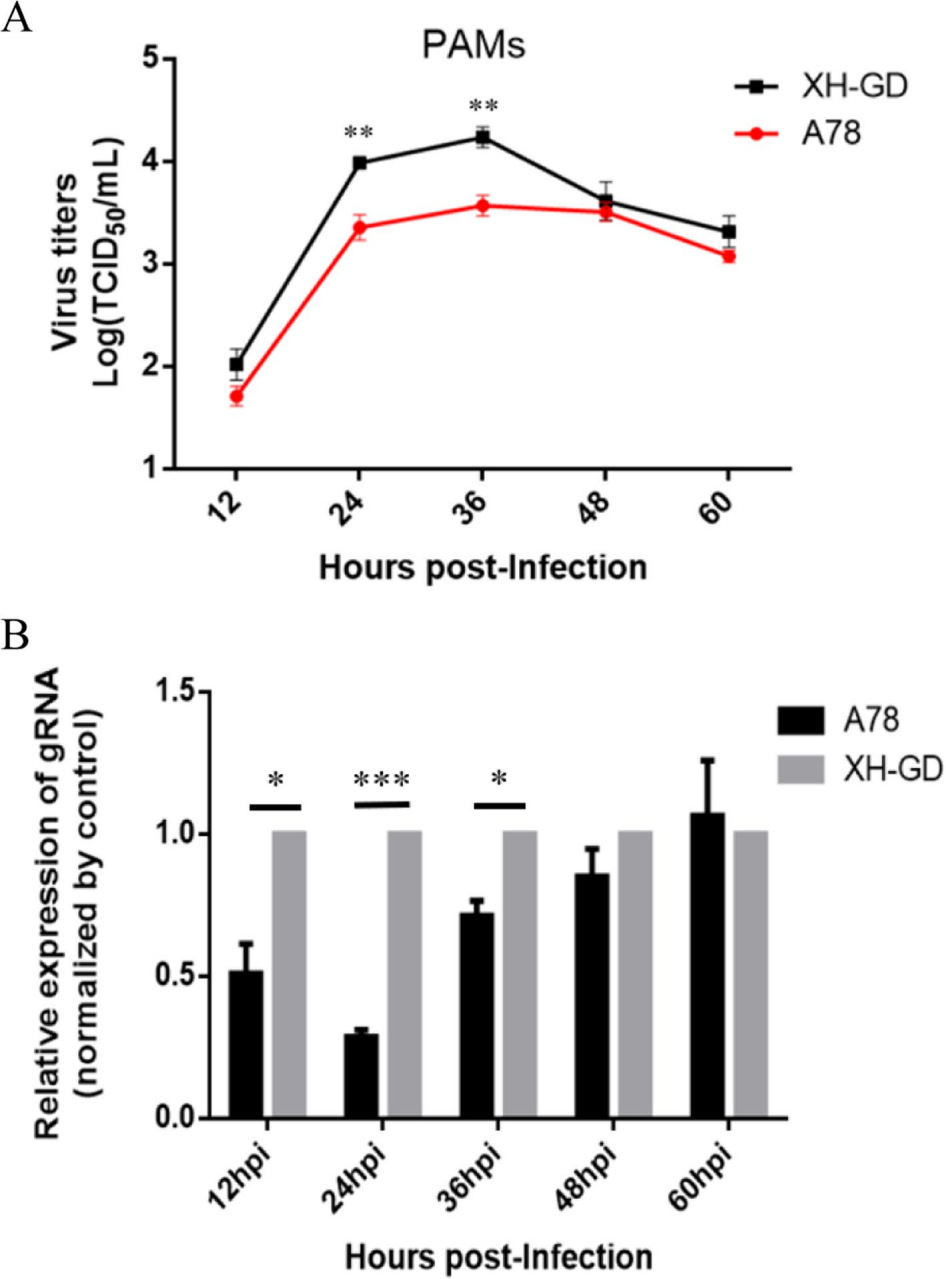
Growth characterization of the A78 in PAMs **(A):** The multistep kinetics of the mutated viruses in PAMs, the cells were infected 0.01 MOI PRRSV, the supernatants were collected at various time points and titrated. The viral titres were calculated by the Reed-Muench method; **(B):** The gRNA qPCR results of the A78 and XH-GD in PAMs. The viral RNA was extracted and subjected to qPCR analysis, and the change in gRNA levels compared with β-actin was determined. Each data point represents the mean value of triplicates. (*, *P* < 0.05; **, *P* < 0.01; ***, *P* < 0.001 in comparison with the XH-GD group)

Finally, we used the primary PAMs to check the results. The cells were infected 0.01MOI XH-GD or A78, the multistep growth kinetics showed that the titer of A78 was significantly lower than XH-GD from 24 to 36 hpi (Fig. 5A). The qPCR results suggest the the gRNA level of XH-GD was significantly higher than A78 from 12 to 36 hpi (Fig. 5B).These results demonstrated that the mutation of amino acid 78 from serine to alanine could impair viral replication.

### The mutated S78A of the N protein could affect viral sgmRNA transcription

**Fig. 6:**
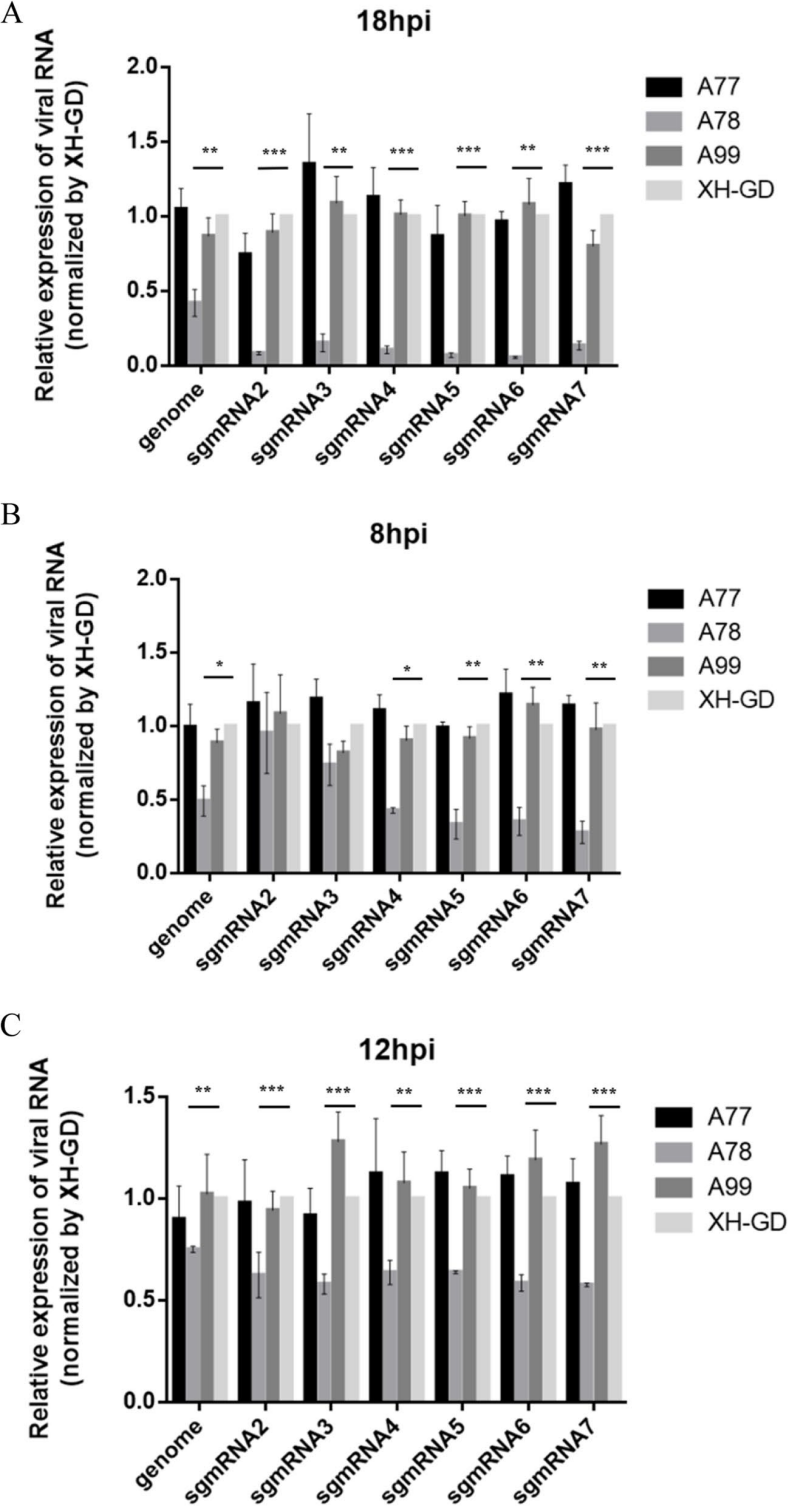
The mutation of amino acid 78 from serine to alanine could inhibit viral sgmRNA transcription. **A**: BHK-21 cells were transfected with 2 μg PRRSV infected clones. At 18hpi, the samples were collected. The relative viral RNA level, compared with XH-GD, was determined. **B-C**: Marc-145 cells were infected with the PRRSV at an MOI of 0.5, and the cells were collected at 8 hpi and 12 hpi. The relative viral RNA level, compared with XH-GD, was determined. The data shown represent the mean ± SD (*n* = 3). (*, *P* < 0.05; **, *P* < 0.01; ***, *P* < 0.001 in comparison with the XH-GD group)

**Fig. 7:**
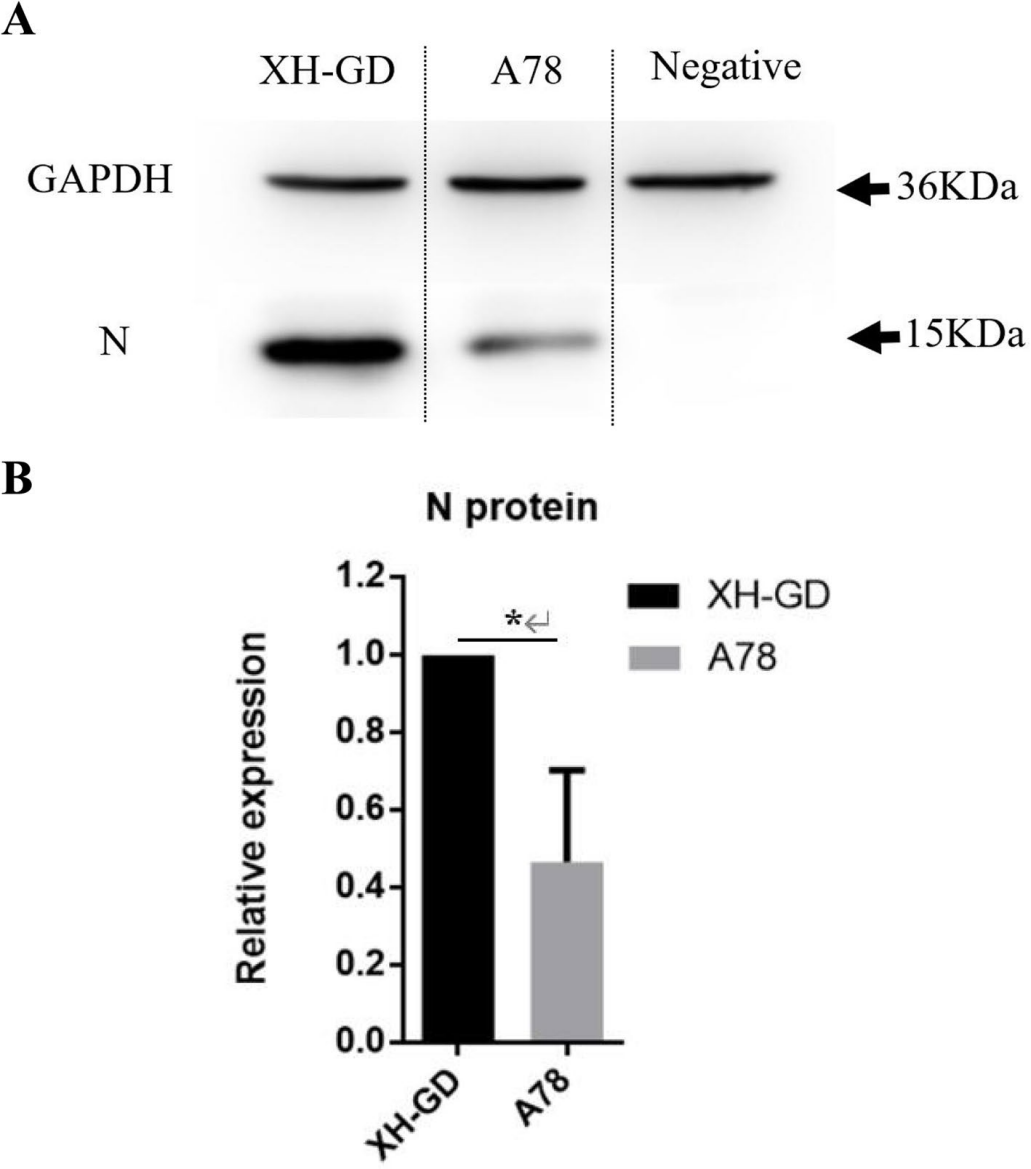
N protein expression level of A78 and XH-GD. **A** Marc-145 cells were infected with XH-GD or A78 at an MOI of 0.5. At 12 hpi, the cells were lysed, and the N protein expression of PRRSV was detected by western blotting. GAPDH was chosen as the control. **B** The pixel intensity was quantified using ImageJ (version 1.8.0). The data shown represent the mean ± SD (*n* = 3). (*, *P* < 0.05; **, *P* < 0.01; ***, *P* < 0.001 in comparison with the XH-GD group)

Participation in viral RNA transcription is another function of the N protein [24]. To investigate whether mutated serine^78^ can change viral RNA transcription, 2 μg of the full-length infectious clones (A78, A99 and XH-GD) were transfected into BHK-21 cells. At 18hpi, the samples were collected for qPCR. There is no difference between the A99 and XH-GD in the sgmRNAs level. However, the results showed that the sgmRNAs of A78 were lower than that of the control group (Fig. 6a). To further verify this result, 0.5MOI A78, A99 and XH-GD were cultivated in Marc-145 cells, and the samples were collected at the early infection stage (8 hpi and 12 hpi). The qPCR results showed that, except for the sgmRNA2 and sgmRNA3 at 8 hpi, the sgmRNAs of A78 were significantly reduced compared with those of the control group (Figs. 6B, 6C). As expected, compared with XH-GD, the N protein level of A78 was decreased by approximately 46.5% at 12 hpi (Fig. 7) (the images have been cropped, the full-length blots are presented in Supplementary Figure S1). This result suggests that the mutation to serine^78^ of the N protein changed the relative accumulation of sgmRNAs.

### The mutated S78A of the N Protein were stable in PRRSV-2 genome

**Fig. 8:**
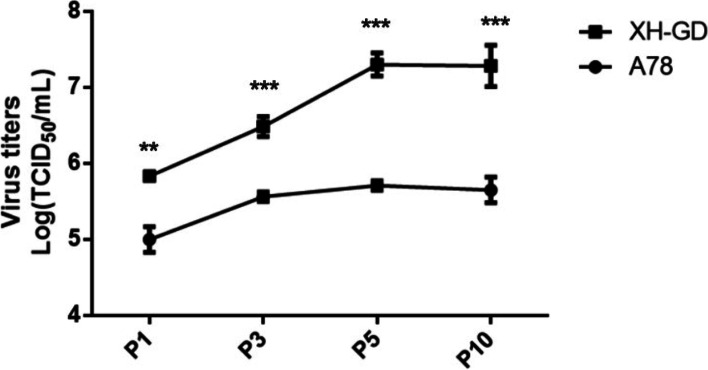
Viral titres of XH-GD and A78 cells at different passages. At each passage, Marc-145 cells were infected with 0.1 MOI PRRSV, and at 48 hpi, the supernatant was collected to determine the virus titre. The TCID_50_ was calculated by the Reed-Muench method. Each data point represents the mean value of triplicates. (*, *P* < 0.05; **, *P* < 0.01; ***, *P* < 0.001 in comparison with the XH-GD group)

**Fig. 9:**
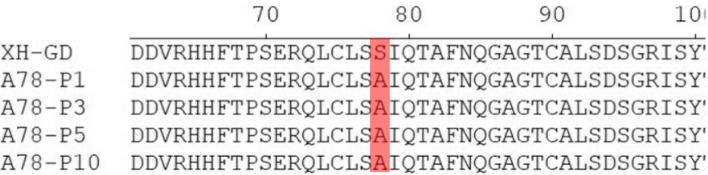
Amino acid analysis of the different passages of A78 cells

To study the stability of the A78 mutants, Marc-145 cells were serially passaged with A78 and XH-GD. P1, P3, P5, and P10 were selected, and sequencing analysis of the N protein showed that the S78A mutations were stable. Then, 0.1 MOI viruses (P1, P3, P5 and P10) were cultivated in Marc-145 cells. The samples were collected at 48 hpi to compare the virus titres. The results showed that the titres of A78 were always lower than those of XH-GD (Fig. 8). The sequence showed that the S78A mutation could stably exist in the PRRSV-2 genome (Fig. 9). These results further confirmed the important role of S78 of the N protein in PRRSV replication.

## Discussion

PRRSV is one of the most important viruses for the swine industry and has caused significant economic losses. Since viable treatments are not available yet, vaccines are still the primary means to protect pigs from PRRSV infections [7]. Attenuating the virulence of PRRSV is the key step in developing an attenuated vaccine. Therefore, it is significant to study the virus replication mechanism and pathogenic mechanism. Many studies have proved that mutating amino acids of the proteins, such as the residues 283, 526, and 627 of PB2 of the AIV and residues 452,484 of the S protein of the SARS-CoV-2, could change protein functions or virus replication ability and virulence [25]. In PRRSV, it has been proven that aspartic^185^ of the Nsp4 could regulate the capacity to antagonize IFN-I expression and lead to a slower replication rate for PRRSV [14]; Mutations to serine^519^, threonine^544^, threonine^586^ and serine^592^ of Nsp9 have proved to affect the replication ability and virulence of high pathogenicity (HP) –PRRSV [16]. Serine^918^ of the Nsp2 has proved to regulate virus production [26].

As a nucleocapsid protein of the PRRSV, the N protein plays an important role in the viral life cycle. The N protein has only 123 or 128 amino acids, and some amino acids were proved that could affect the function of the N protein [27]. Previous studies have shown that the three serine sites of the N protein are important for N protein functions or viral replication. This suggests that mutating the serine of the N protein may be an effective way to change N protein functions or reduce PRRSV viral replication [19]. In this study, the remaining serine N proteins were individually substituted with alanine, and ten plasmids were constructed. The plasmids were transfected into PAM cells, and the qPCR results showed that the IL-10 mRNA levels of pCA-78 and pCA-99 were significantly lower than those of the control group. This result suggested that mutations to serine^78^ and serine^99^ can impair N protein functions.

Four of the serine sites (serine^36^, serine ^105^, serine ^120^ and serine ^122^) of the N protein have been proven to not affect the recovery of PRRSV-2, but some of them impair viral replication ability [18].The IFA result showed that the S78A or S99A mutations did not affect viral viability or infectivity. Then, the replication abilities of the different strains were compared by multistep growth kinetics and qPCR. We found that the titres of A78 were significantly lower than those of XH-GD from 36 to 60 hpi. This suggests that the mutated serine 78 could impair PRRSV-2 replication in vitro. Besides, we used a reverse genetic technique to mutate the rest of the serine sites. The results demonstrated that the other serine site did not affect viral viability, infectivity or replication ability (supplementary file Figure S3).

Since A36 and A99 showed multistep growth kinetics similar to those of XH-GD, at meantime, we also compared the level of IL-10 mRNA production of A78 with XH-GD in the primary PAMs, we found there were no significantly different between A78 and XH-GD (supplementary file Figure S4). It means that inhibiting the ability of the N protein to induce IL-10 did not lead to the downregulation of PRRSV replication. Poor growth ability could have other mechanisms. Previous studies showed that mutated serine ^918^ of the Nsp2 of PRRSV-1 could regulate the expression level of viral genomes and sgmRNAs. The N protein could also interact with viral RNA or other proteins and participate in viral genomic transcription. Therefore, we detected the viral RNA level at different time points by qPCR, and the mutation of serine 78 of the N protein changed the relative accumulation of sgmRNAs. Finally, the passage test proved that the S78A mutation could stably exist in the PRRSV-2 genome. It suggested that this mutation may be used as a molecular marker.

Coronaviruses and arteriviruses have similar discontinuous transcription mechanisms [28]. As previous studies have shown, the functions and structure of the PRRSV N protein were similar to those of the N protein of the *coronavirus *[29]. In the case of SARS-CoV, mutation of the serine sites modified by phosphorylation can change the proportion of sgmRNAs and gRNA by DEAD-box RNA helicase 1 (DDX1) [30]. The N protein of PRRSV was found to connect with DDX5 or DHX9 [24, 31], which were found to regulate PRRSV sgmRNA transcription. Therefore, we speculated that a mutation to serine 78 could affect the N protein interaction with DDX5 or DHX9 and affect viral genome transcription. In addition, we noticed that serine 78 of the N protein has a similar function to serine^105^ and serine^120^, which were proven to be modified by phosphorylation. These results indicated that serine 78 may be a phosphorylation site similar to the other serine sites. However, these hypotheses still need the further investigations.

## Conclusion

In this study, our results showed that serine^78^ and serine^99^ can affect the N-induced expression of IL-10 mRNA in transfected PAMs; mutations of S78A of the N protein impaired PRRSV-2 replication in vitro and sgmRNA transcription. However, the mechanisms need further investigation. These studies will be helpful for understanding the replication and transcription mechanisms of PRRSV.

## Methods

### Cells and viruses

Marc-145 cells (China Center for Type Culture Collection, CCTCC, China) and BHK-21 cells (China Center for Type Culture Collection, CCTCC, China)were cultivated in Dulbecco’s modified Eagle’s medium DMEM (BI, Israel) supplemented with 10% foetal bovine serum (FBS, Gibco, USA) at 37 °C under 5% CO_2_. Porcine alveolar macrophages (,PAMs,ATCC,3D4/2,USA) or primary PAMs were cultivated in an RPMI 1640 medium (Gibco, USA) supplemented with 10% FBS (Gibco, USA) at 37 °C under 5% CO_2_. The virus (XH-GD, GenBank: EU624117.1) was cultivated in Marc-145 cells in DMEM containing 2% FBS.

### Plasmid construction

PCR was used to prepare the different N genes, and the primers are shown in Table 1. Then, the N genes were inserted into the pCAGGS-MCS expression plasmid (ID: G0609), and ten plasmids (pCA-A36, pCA-A70, pCA-A77, pCA-A78, pCA-A93, pCA-A95, pCA-A99, pCA-A105, pCA-A122 and pCA-XH-GD) were constructed. Two micrograms of plasmid were transfected into PAM cells (3D4/2) by Lipofectamine™ 3000 (Invitrogen, USA) as previously reported [18]. The empty vector was picked as the control group.

**Table 1:** The primer of constructing the plasmid construction and PRRSV infection clones

Name	Sequence (5’-3’)
N-pca-F-EcoRI	CC*GAATTC*ATGCCAAATAACAACGGCAAGC
N-pca-R-Xhol	TT*CTCGAG*TCATGCTGAGGGTGATGCTG
N-pca-R-122	TT*CTCGAG*TCATGCCGGCGGTGATGCTG
XmaI-F	CACGTCGAAAGTGCCGCG
KpnI-122-R	GATGTGCTGCAAGGCGAT
BbvCI -R	CCGCATGGTTCTCGCCAATTA
N36A-F	CATCGCCCAACAAAACCAGGCCAGAGGCAA
N36A-R	CCCCGGTCCCTTGCCTCTGGCCTGGTTTTG
N70A-F	GGCATCACTTTACCCCTGCTGAGCGGCAAT
N70A-R	CAGACACAATTGCCGCTCAGCAGGGGTAAA
N77A-F	GTGAGCGGCAATTGTGTCTGGCGTCGATCC
N77A-R	GAAGGCAGTCTGGATCGACGCCAGACACAA
N78A-F	GGCAATTGTGTCTGTCGGCGATCCAGACTG
N78A-R	CTGATTGAAGGCAGTCTGGATCGCCGACAG
N93A-F	GCGCTGGAACTTGTGCCCTGGCAGATTCAG
N93A-R	AACTTATCCTCCCTGAATCTGCCAGGGCAC
N95A-F	GGAACTTGTGCCCTGTCAGATGCAGGGAGG
N95A-R	AGTGTAACTTATCCTCCCTGCATCTGACAG
N99A-F	GTCAGATTCAGGGAGGATAGCTTACACTGT
N99A-R	AAACTAAACTCCACAGTGTAAGCTATCCTC
N122A-F	CCGCGCCACAGCATCACCCGCAGCATGATG
N122A-R	GAATGCCAGCCCATCATGCTGCGGGTGATG

### The construction of PRRSV infectious cDNA clones and recombinant virus rescue

The PRRSV infectious clones were similar to those from a previous report (supplementary file Figure S1) [18], the target segments were the product of fusion PCR (Table1). Then, using digestion and T4 ligase (Thermo, USA), the N genes of POK-XH-GD were replaced, and nine infectious clones were constructed. The full-length infectious clones were transfected into BHK-21 cells or Marc-145 cells by Lipofectamine™ 3000 (Invitrogen, USA) according to the manufacturer’s instructions. After 48 h, the cells were collected as the primary passage. The mutated viruses were named A78, A99, A105. Then, the viruses were cultivated in Marc-145 cells serially for three passages (P1-P3).

### Quantitative PCR

Total RNA was extracted by a commercial RNA extraction kit according to the manufacturer’s instructions (Fastagen, China). One microgram of RNA was used to synthesize cDNA with a reverse transcription kit (Takara, Japan). qPCR was carried out by a CFX96™ Real-Time System (Bio–Rad, USA). According to the TB Green® Premix Ex Taq™ (Tli RNaseH Plus) manual (Takara, Japan), the qPCR program parameters were 95 °C for 30 s followed by 45 cycles at 95 °C for 5 s and 60 °C for 30 s. The primers are listed in Table 2. The β-actin gene was the control. Relative mRNA expression was calculated by the 2^−ΔΔ^CT method.

**Table 2:** The primer used in qPCR

Name	Sequence (5’-3’)
IL10-F	TTCAAACGAAGGACCAGATG
IL10-R	CACAGGGCAGAAATTGATGA
β-actin-F	GCGGGACATCAAGGAGAAG
β-actin-R	AGGAAGGAGGGCTGGAAGAG
gRNA-F	CCCTCCATGCCAAACTACCAC
gRNA-R	TTGTCTTCTTTGGGTCCGTCT
Leader-F	CACCTTGCTTCCGGAGTTG
sgmRNA2-R	CAGCCAACCGGCGATTGTGAA
sgmRNA3-R	GCAAAGCGGGCATACCGTGT
sgmRNA4-R	ACGAAGTCTGATGCTGCGGTG
sgmRNA5-R	CTGGCGTTGACGAGCACAGCA
sgmRNA-6-R	CATCACTGGCGTGTAGGTAATGGA
sgmRNA7-F	CCCGGGTTGAAAAGCCTCGTGT
sgmRNA7-R	GGCTTCTCCGGGTTTTTCTTCCTA

### Immunofluorescence assay (IFA)

Marc-145 cells were cultivated in 6-well plates and infected with PRRSV at an MOI of 0.1. At 48 h postinfection (hpi), the cells were treated with paraformaldehyde for 30 min at 4 °C. Then, the Marc-145 cells were fixed with 0.1% Triton X-100 for 1 h and blocked with 5% skim milk for 2 h. Mouse anti-N-protein antibodies (Median, South Korea) (1:400 dilution) were incubated with the cells at 4 °C overnight. Subsequently, the cells were washed five times with PBS and incubated with fluorescein isothiocyanate (FITC)-conjugated anti-mouse IgG (1:100 dilution) as the secondary antibody for 1 h at 37 °C. Finally, fluorescence was observed by fluorescence microscopy (ECHO, USA).

### Multistep growth curve

Marc-145 cells were infected with the virus at a multiplicity of infection (MOI) of 0.1. Supernatants were collected at certain time points (12 h, 24 h, 36 h, 48 h, 60 h, and 72 h). Then, the Marc-145 cells cultivated in 96-well plates were used to measure the titres. The results were calculated using the Reed-Muench method.

### Western blotting

The Marc-145 cells were infected with 0.5MOI PRRSV. At 12 hpi, the cells were lysed with RIPA lysis buffer (Beyotime, China). The samples were subjected to sodium dodecyl sulfate (SDS, 15%)-polyacrylamide gel electrophoresis (PAGE). Immunoblot analysis was performed as previously reported [18]. Mouse anti-N-protein antibodies (Median, South Korea,SOW17) (1:1000 dilution) and monoclonal GAPDH antibodies (Beyotime, China) were used as primary antibodies, and goat anti-mouse IgG with horseradish peroxidase (HRP) (Beyotime, China) was used as the secondary antibody. The membranes were analysed by Biosystems C280 (Azure, USA). Pixel intensity was quantified using ImageJ (version 1.8.0).

## Data analysis

All data were analysed as the means ± standard deviation (SD) of three independent experiments. Statistical analyses were performed using SPSS software (version 21.0). Independent-sample t-tests were used to evaluate the differences among the groups, and *p* < 0.05, *p* < 0.01, and *p* < 0.001 were considered statistically significant at different levels.

## Supplementary Information

**Additional file 1:** **Figure S1.**The schematic diagram of the PRRSV infectious cDNA clones. **Figure S2.**The full-length western-blots of N proteinexpression level of A78 and XH-GD. **Figure S3. **The rest serine sitesdid not affect viral viability, infectivityor replication ability.**(A)**: IFA results. The Marc-145 cells were infectedwith the PRRSV at 0.1MOI. At 48 hpi, the cells were fixed with 4%paraformaldehyde and PBS. Then, the cells were incubated with the N monoclonalantibody, followed by goat anti-mouse IgG (H+L), which was modified by fluoresceinisothiocyanate (FITC). The nuclei were stained with DAPI (Scale bars are 90 um); **(B)**Growth characterization of the viruses. Themultistep kinetics of the mutated viruses. Marc-145 cells were infected withPRRSV at an MOI of 0.1. Thesupernatants were collected at various time points and titrated. The viraltitres were calculated by the Reed-Muench method. **Figure S4. **Level of IL-10 mRNA production of A78, the result wascalculated after normalization to XH-GD. The data shown represent the mean ±SD (*n*=3).

## Data Availability

The datasets supporting the conclusions of this article are included within the article.

## Abbreviations

N protein: Nucleocapsid protein
PRRS: Porcine reproductive and respiratory syndrome
PRRSV: Porcine reproductive and respiratory syndrome virus
PAMs: Porcine alveolar macrophages
qPCR: Quantitative polymerase chain reaction
IL-10: Interleukin-10
gRNA: Genome RNA
sgmRNA: Subgenomic RNA
IFA: Indirect Immunofluorescence Assay
IFN-I: Type I interferon
Nsp4: Nonstructural protein 4
Nsp9: Nonstructural protein 9
MOI: Multiplicity of Infection
DMEM: Dulbecco’s modified Eagle’s Medium
FBS: Fetal bovine sera
RPMI 1640: Roswell Park Memorial Institute 1640 medium
hpi: Hours post-infection
PBS: Phosphate Buffer Saline
FITC: Fluorescein isothiocyanate
DDX1: DEAD-box RNA helicase 1
DDX5: DEAD-box RNA helicase 5
TRS: Transcription regulating sequence
SD: Standard deviations
SARS-CoV: Severe acute respiratory syndrome coronavirus
TCID_50_: Tissue culture infective dose 50
