# Supplementary material for: A novel amino acid site of N protein could affect the PRRSV-2 replication by regulating the viral RNA transcription
Source: BMC Vet Res. 2022 May 11;18:171. doi: 10.1186/s12917-022-03274-9 (PMC9092334; doi:10.1186/s12917-022-03274-9)

**FIGURE S1** The schematic diagram of the PRRSV infectious cDNA clones.

5’UTR

3’UTR

*Xma* I

*Bbvc* I

*AfI* II

*Bam* HI

*Hind* III

ORF1a

1b

2a

2b

3

4

5

6

7

A

B

C

D

5a

pokq

*Not* I

*Kpn* I

**FIGURE S2** The full-length western-blots of N protein expression level of A78 and XH-GD.

A78

Negative

XH-GD


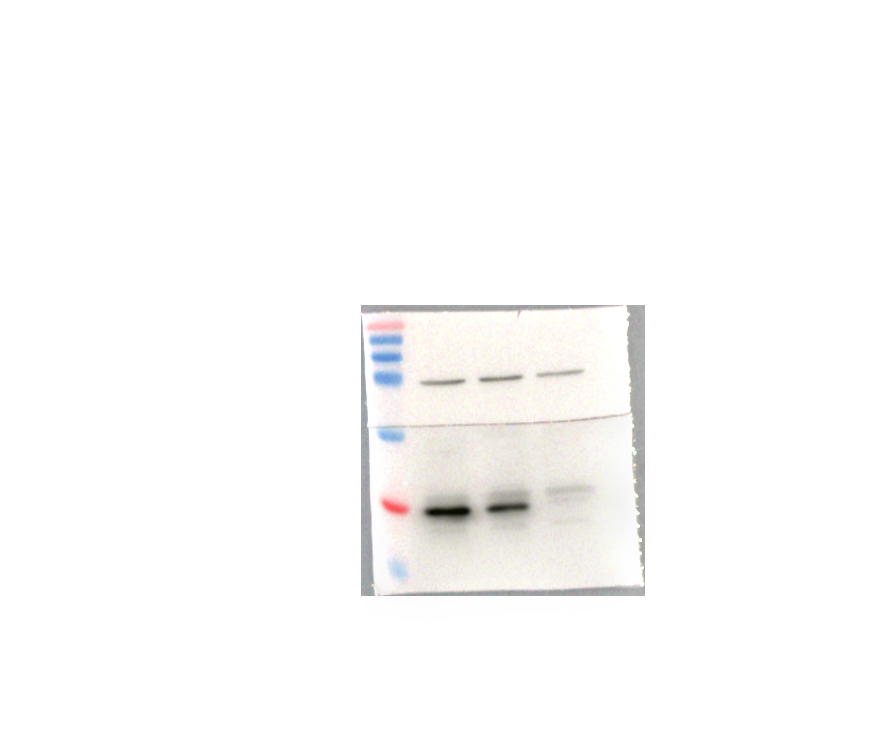


N

GAPDH

**FIGURE S3** The rest serine sites did not affect viral viability, infectivity or replication ability.**(A)**: IFA results. The Marc-145 cells were infected with the PRRSV at 0.1MOI. At 48 hpi, the cells were fixed with 4% paraformaldehyde and PBS. Then, the cells were incubated with the N monoclonal antibody, followed by goat anti-mouse IgG (H+L), which was modified by fluorescein isothiocyanate (FITC). The nuclei were stained with DAPI (Scale bars are 90 um); **(B)**Growth characterization of the viruses. The multistep kinetics of the mutated viruses. Marc-145 cells were infected with PRRSV at an MOI of 0.1. The supernatants were collected at various time points and titrated. The viral titres were calculated by the Reed-Muench method

**
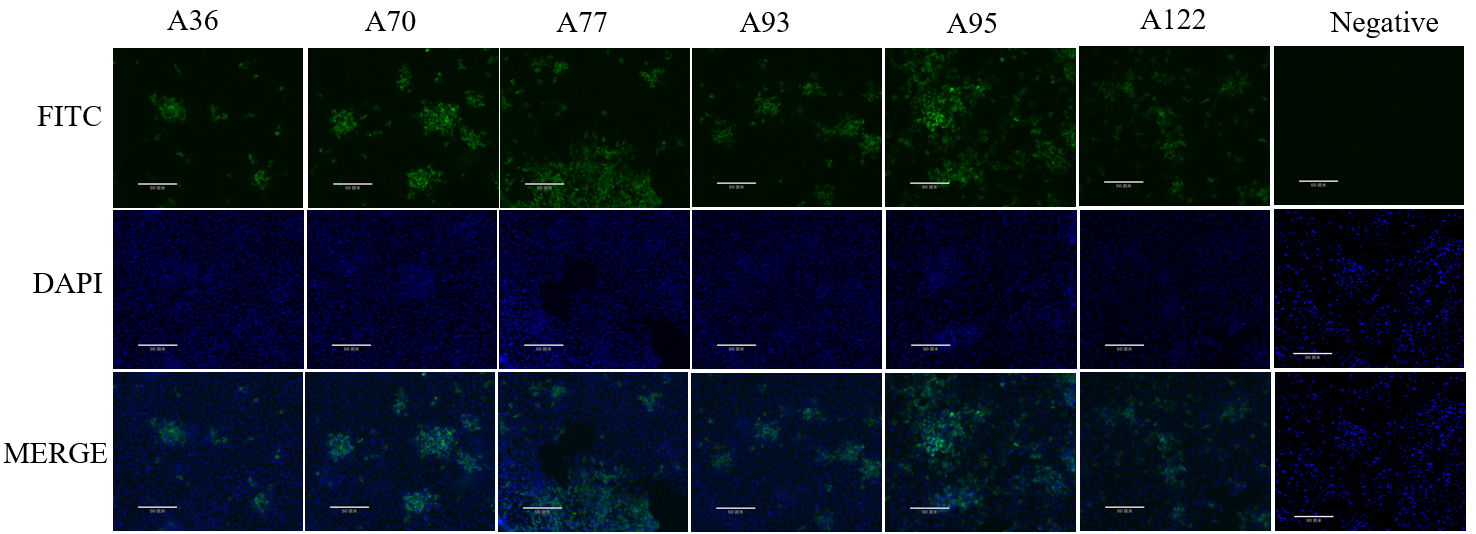
A**

**B**


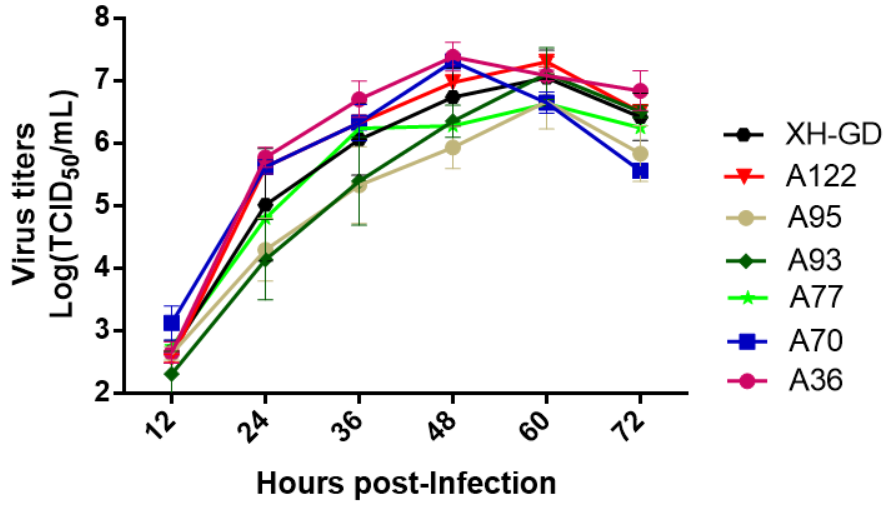


**FIGURE S4** Level of IL-10 mRNA production of A78, the result was calculated after normalization to XH-GD. The data shown represent the mean ± SD (*n*=3).


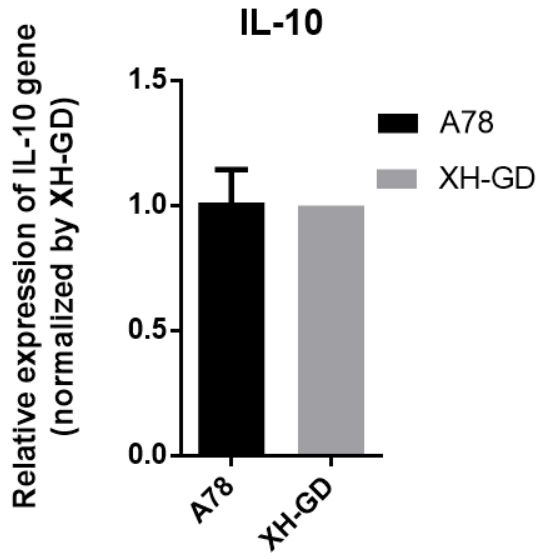

Supplement: Supplementary file 1 — Additional file 1: Figure S1.The schematic diagram of the PRRSV infectious cDNA clones. Figure S2.The full-length western-blots of N proteinexpression level of A78 and XH-GD. Figure S3. The rest serine sitesdid not affect viral viability, infectivityor replication ability.(A): IFA results. The Marc-145 cells were infectedwith the PRRSV at 0.1MOI. At 48 hpi, the cells were fixed with 4%paraformaldehyde and PBS. Then, the cells were incubated with the N monoclonalantibody, followed by goat anti-mouse IgG (H+L), which was modified by fluoresceinisothiocyanate (FITC). The nuclei were stained with DAPI (Scale bars are 90 um); (B)Growth characterization of the viruses. Themultistep kinetics of the mutated viruses. Marc-145 cells were infected withPRRSV at an MOI of 0.1. Thesupernatants were collected at various time points and titrated. The viraltitres were calculated by the Reed-Muench method. Figure S4. Level of IL-10 mRNA production of A78, the result wascalculated after normalization to XH-GD. The data shown represent the mean ±SD (n=3). [file 12917_2022_3274_MOESM1_ESM.docx]
